# Identification of maize kernel varieties based on interpretable ensemble algorithms

**DOI:** 10.3389/fpls.2025.1511097

**Published:** 2025-02-12

**Authors:** Chunguang Bi, Xinhua Bi, Jinjing Liu, Hao Xie, Shuo Zhang, He Chen, Mohan Wang, Lei Shi, Shaozhong Song

**Affiliations:** ^1^ Institute for the Smart Agriculture, Jilin Agricultural University, ChangChun, China; ^2^ College of Information Technology, Jilin Agricultural University, Changchun, China; ^3^ Institute of Science and Technology, Changchun Humanities and Sciences College, Changchun, Jilin, China; ^4^ Jilin Zhongnong Sunshine Data Co., Changchun, China; ^5^ School of Data Science and Artificial Intelligence, Jilin Engineering Normal University, Changchun, China

**Keywords:** maize kernel, variety identification, stacking ensemble model, multimodal data, differential evolutionary algorithm, SHAP value

## Abstract

**Introduction:**

Maize kernel variety identification is crucial for reducing storage losses and ensuring food security. Traditional single models show limitations in processing large-scale multimodal data.

**Methods:**

This study constructed an interpretable ensemble learning model for maize seed variety identification through improved differential evolutionary algorithm and multimodal data fusion. Morphological and hyperspectral data of maize samples were extracted and preprocessed, and three methods were used to screen features, respectively. The base learner of the Stacking integration model was selected using diversity and performance indices, with parameters optimized through a differential evolution algorithm incorporating multiple mutation strategies and dynamic adjustment of mutation factors and recombination rates. Shapley Additive exPlanation was applied for interpretable ensemble learning.

**Results:**

The HDE-Stacking identification model achieved 97.78% accuracy. The spectral bands at 784 nm, 910 nm, 732 nm, 962 nm, and 666 nm showed positive impacts on identification results.

**Discussion:**

This research provides a scientific basis for efficient identification of different corn kernel varieties, enhancing accuracy and traceability in germplasm resource management. The findings have significant practical value in agricultural production, improving quality management efficiency and contributing to food security assurance.

## 1 Introduction

The problem of global food security is becoming increasingly serious, and maize, as one of the major food crops globally (Tyczewska et al., 2018), shoulders the important mission of securing human food supply. Kernel quality of maize not only affects germination and growth and development, but also directly relates to the final yield and economic benefits (Sehgal et al., 2018). Therefore, accurate and rapid identification of maize kernels not only helps to improve the efficiency of agricultural production, but also ensures that high-quality kernels reach the market and reduces the waste of resources and economic losses (Yang et al., 2017; Wang D. et al., 2021; Zhang et al., 2022). Although traditional identification methods such as high performance liquid chromatography (HPLC), protein electrophoresis and DNA molecular labelling have high accuracy, these methods generally have significant drawbacks such as being destructive, costly and time-consuming (Zareef et al., 2021; Zhang et al., 2024), so it is important to develop rapid, non-destructive and economical methods for seed variety identification.

In recent years, with the rapid development of information technology, the application of multimodal data has gradually become a trend in the identification of maize kernel varieties (Zhou et al., 2017). Multimodal data refers to multidimensional information acquired through a number of different perceptual means or data sources. Image data can capture the appearance characteristics of seeds, including shape, color, and surface texture, but the appearance characteristics are often not sufficient to comprehensively reflect the intrinsic quality differences of seeds (Khojastehnazhand and Roostaei, 2022). Hyperspectral technology reveals the internal chemical composition and physical structure of seeds by collecting reflectance spectral data in multiple spectral bands, providing richer information for seed identification (Hu et al., 2022). Researchers have proposed various improvements to address the limitations of a single learner. **Table 1** presents a performance comparison of different techniques in seed variety classification. While deep learning methods have demonstrated exceptional performance in image-based classification tasks due to their powerful feature learning capabilities, they require larger training datasets, longer training periods, and exhibit lower interpretability compared to traditional machine learning approaches. Notably, the fusion of image and spectral data has shown significant advantages, achieving 97.7% accuracy in ten-class maize classification tasks. These results indicate that single data sources often overlook crucial feature information, whereas the fusion of multiple features can enhance feature representation through complementary effects. Consequently, researchers have started investigating the integration of information from multiple feature types to enhance classification and discrimination tasks. Huang et al. (2016a) combined morphological and spectral features for maize seed classification, achieving an accuracy of 92.65% using a least squares support vector machine classifier. Li et al. (2023) combined morphological and hyperspectral features to predict cotton seed vigor using a modified one-dimensional CNN model and obtained a correlation coefficient of 0.9427 after fusion of spectral and image features. Yang et al. (2015) utilized hyperspectral imaging combining spectral and image features and used SVM model for identification with up to 98.2% accuracy. Scholl et al. (2021) proposed a deep learning method that fuses hyperspectral, LiDAR and RGB data to significantly improve the accuracy of crop identification. These studies demonstrate the great potential of multimodal data in maize kernel variety identification, when the capability of single-modal data is limited, multimodal fusion can effectively make up for its shortcomings.

**Table 1:** Classification results of different technologies.

Sorting Technology	Data Type	Number of Varieties and Categories	Model	Accuracy (%)	Ref
Machine learning	Hyperspectral	five types of wheat	ELM	86.26	(Bao et al., 2019)
	NIR+HSI	seven types of cotton	PLS-DA, LR, SVM	80	(Zhu et al., 2019)
	RGB	five types of maize	MLP, LDA, SVM etc.	93	(Xu et al., 2021)
Deep learning	NIRS	four years of cotton seeds	CNN, RNN, LSTM, etc.	93.5	(Duan et al., 2021)
	HSI	eight types of wheat seeds	CNN	95.65	(Zhao et al., 2022)
	RGB	six types of maize	ResNet50	91.23	(Li et al., 2024)
	RGB+NIRS	ten types of maize	BP Neural Network	97.7	(Yang and Hu, 2023)

Meanwhile, the effective integration of high-dimensional and complex data is still a great challenge. With the rapid development of artificial intelligence, machine learning methods have been widely applied in agricultural research, showing great potential in crop yield prediction (Guo et al., 2021, 2022, 2023), crop phenotyping (Ubbens and Stavness, 2017; Mochida et al., 2019; Zheng et al., 2021), growth monitoring (Singh et al., 2016; Moysiadis et al., 2023; Aierken et al., 2024) and variety identification (Kurtulmuş and Ünal, 2015; Tu et al., 2022). Traditional single algorithms have limited learning capabilities and are prone to overfitting when dealing with multimodal data (Duan et al., 2024). Ensemble learning techniques are gradually being introduced to further drive model performance optimization (Wang L. et al., 2021). In agricultural applications, Umamaheswari and Madhumathi (2024) developed a Stacking ensemble model for crop yield prediction by combining multiple machine learning algorithms, which significantly improved prediction accuracy compared to single models. Wang et al. (2024) proposed an innovative Stacking framework that integrated crop simulation models with machine learning methods to estimate pakchoi dry matter yield, demonstrating the advantages of ensemble learning in handling complex agricultural data. Bigdeli et al. (2021) proposed an ensemble deep learning strategy based on CNN-SVM, which significantly improves the accuracy of remote sensing data classification, and the classification accuracy is improved by 2% to 10% compared with the traditional methods. Therefore, this study chooses to solve the problem of maize kernel variety identification from the perspective of ensemble learning. However, in the practical application of machine learning, performance largely depends on the setting of hyperparameters within the model. Therefore, selecting the optimal hyperparameters is the most critical step. The swarm intelligence optimization algorithm can effectively solve nonlinear parameter optimization problems and has strong global search capabilities and adaptability. In recent years, a variety of evolutionary algorithms have been proposed to solve optimization problems, such as the grey wolf optimization algorithm (GWO), the grasshopper optimization algorithm (GOA), and the sparrow search algorithm (SSA). Research has shown that these swarm intelligence algorithms outperform traditional optimization algorithms in many fields, such as speech recognition, image processing, path planning, and data mining. For example, the hybrid particle swarm optimization algorithm proposed by Sudha and Maheswari (2024) improved the accuracy of Mask RCNN to 98.96% in the lung cancer detection task. Shao et al. (2022) applied the sparrow search algorithm to intelligent vehicle classification and achieved an accuracy of 95.83% in multi-modal data analysis. Among the many evolutionary algorithms, this study selected the differential evolution (DE) algorithm as the basic algorithm, mainly based on the following considerations: it has few parameters, is easy to implement and adjust; it has a mature theoretical basis and a wealth of improvement strategies; it performs stably in continuous optimization problems. However, the differential evolutionary algorithm is less efficient in searching when dealing with high-dimensional data, and it is easy to fall into local optimal solutions (Gao et al., 2021). In response to this problem, Zhou et al. (2016) improved the differential evolutionary algorithm through a multi-stage strategy, which significantly improved the quality of solutions and convergence speed in global optimization problems. Liang et al. (2024) proposed a multi-objective differential evolutionary algorithm that improves the quality and diversity of solutions for high-dimensional multimodal multi-objective optimization through a two-population framework. The selection of the base learner is one of the key challenges in optimizing the integrated learning model. Especially when dealing with complex data, considering the performance and diversity of different learners can help improve the generalization ability and robustness of the model.

Interpretability of model decision-making processes becomes particularly important when machine learning models are applied to critical agricultural production decisions such as crop variety identification. Although individual learners in ensemble learning models (e.g., decision trees and logistic regression) are interpretable, understanding the combined decision-making basis of the model remains challenging when dealing with complex multimodal data (Sahlaoui et al., 2021; Zhang et al., 2021; Nordin et al., 2023). Users need to understand these decision bases in order to make reasonable adjustments to agricultural production processes (Ribeiro and dos Santos Coelho, 2020). With the development of explainable artificial intelligence (XAI) technology, the explainability of ensemble learning models has become a hot research topic. Rather than focusing only on the accuracy of the model, users are more concerned about which features play a key role in the decision-making process, which directly affects the quality of decision-making in agricultural management. Charytanowicz (2023) proposed an ensemble machine learning-based framework for wheat grain classification, which achieved 94% classification accuracy and model interpretability through SHAP values, with grain furrow length, grain circumference, and the ratio of embryo area to grain area being the key variables affecting the classification results, especially in the Rosa and Canadian varieties which were the most significant in the Rosa and Canadian varieties. In this context, the design of ensemble learning frameworks with high discriminatory performance and interpretability has become an important research direction in the field of maize kernel variety identification.

To address the above problems, this study proposes a framework combining an improved differential evolutionary algorithm and interpretable ensemble learning for maize seed variety identification based on multimodal data. Specifically, the contributions are mainly in the following aspects:

1. An identification framework combining multimodal data and ensemble learning is proposed: the accuracy of maize seed variety identification is improved by effectively integrating image and hyperspectral data. The limitations of a single data source are overcome through feature-level fusion, and the complementary nature of multimodal data is effectively utilized.

2. Optimized parameter configuration of the ensemble learning model: an improved differential evolutionary algorithm is proposed to optimize the hyperparameter settings of the Stacking ensemble learning model, which enhances the discriminative performance and stability of the model to better adapt to high-dimensional complex data.

3. Interpretable ensemble learning framework is designed: in the ensemble learning model, a new base learner selection and fusion strategy is proposed, which takes into account the discrimination performance and model diversity to ensure the robustness of the discrimination results. Meanwhile, the interpretability of the model is enhanced by introducing the SHAP interpretation mechanism, which makes the final discrimination results not only accurate but also transparent and easy to understand.

## 2 Materials and methods

### 2.1 Material preparation

**Figure 1:**
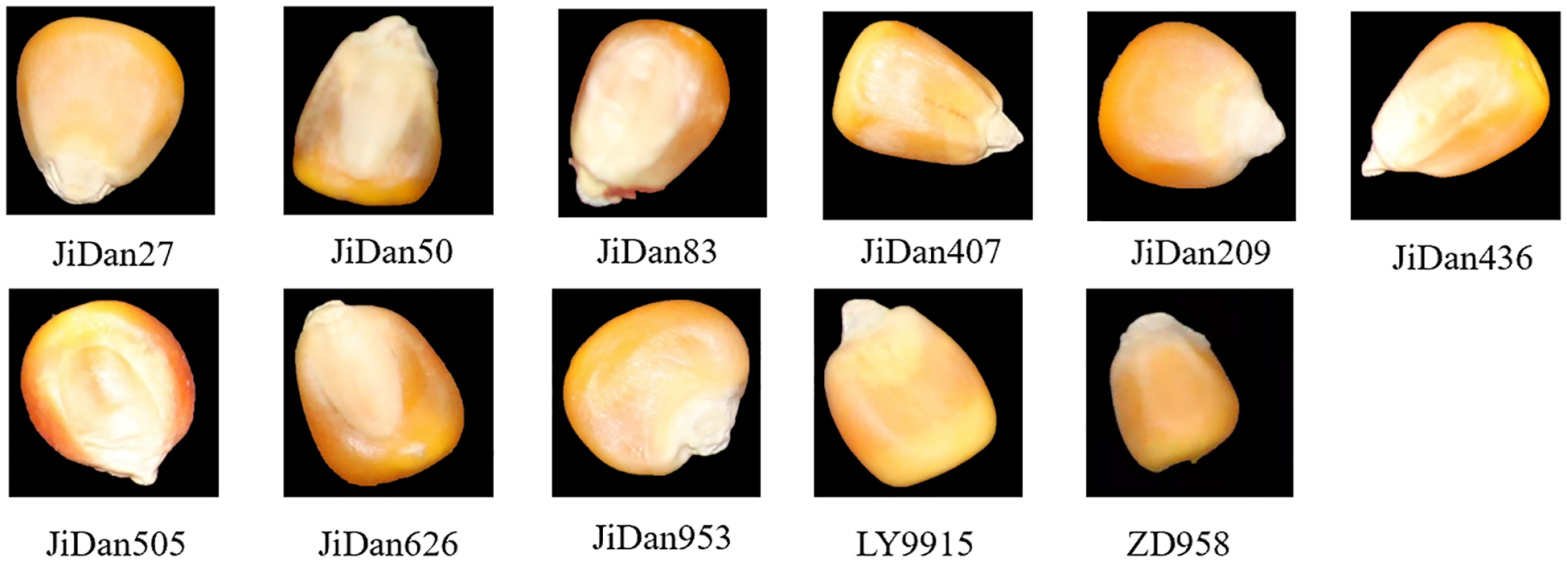
Maize kernel samples.

The maize kernel samples used in this study were provided by Institute of Smart Agriculture at Jilin Agricultural University, and included a total of 11 varieties: JiDan209, JiDan626, JiDan505, JiDan27, JiDan407, JiDan50, JiDan83, JiDan953, JiDan436, LY9915, and ZhengDan958 (**Figure 1**). For model training and evaluation purposes, these varieties were numerically encoded from 0 to 10 in the order listed above. These varieties are representative of the main maize varieties promoted for planting in Jilin Province: the JiDan series of varieties are highly adaptable and have stable yields, and are the dominant varieties in the spring maize zone of Northeast China; ZhengDan958 is a widely adaptable variety in the Huanghuaihai summer maize zone; and LY9915 is an important variety promoted for planting in Northeast China. All selected seeds were yellow in color with a few varieties having a slightly reddish surface. To ensure the purity and integrity of the seed samples, manual screening was conducted during the sampling process to remove broken, insect-damaged and impurity seeds, and finally full and intact seeds were selected. The number of seeds per variety was 1000.

### 2.2 Data acquisition

#### 2.2.1 Image data acquisition

**Figure 2:**
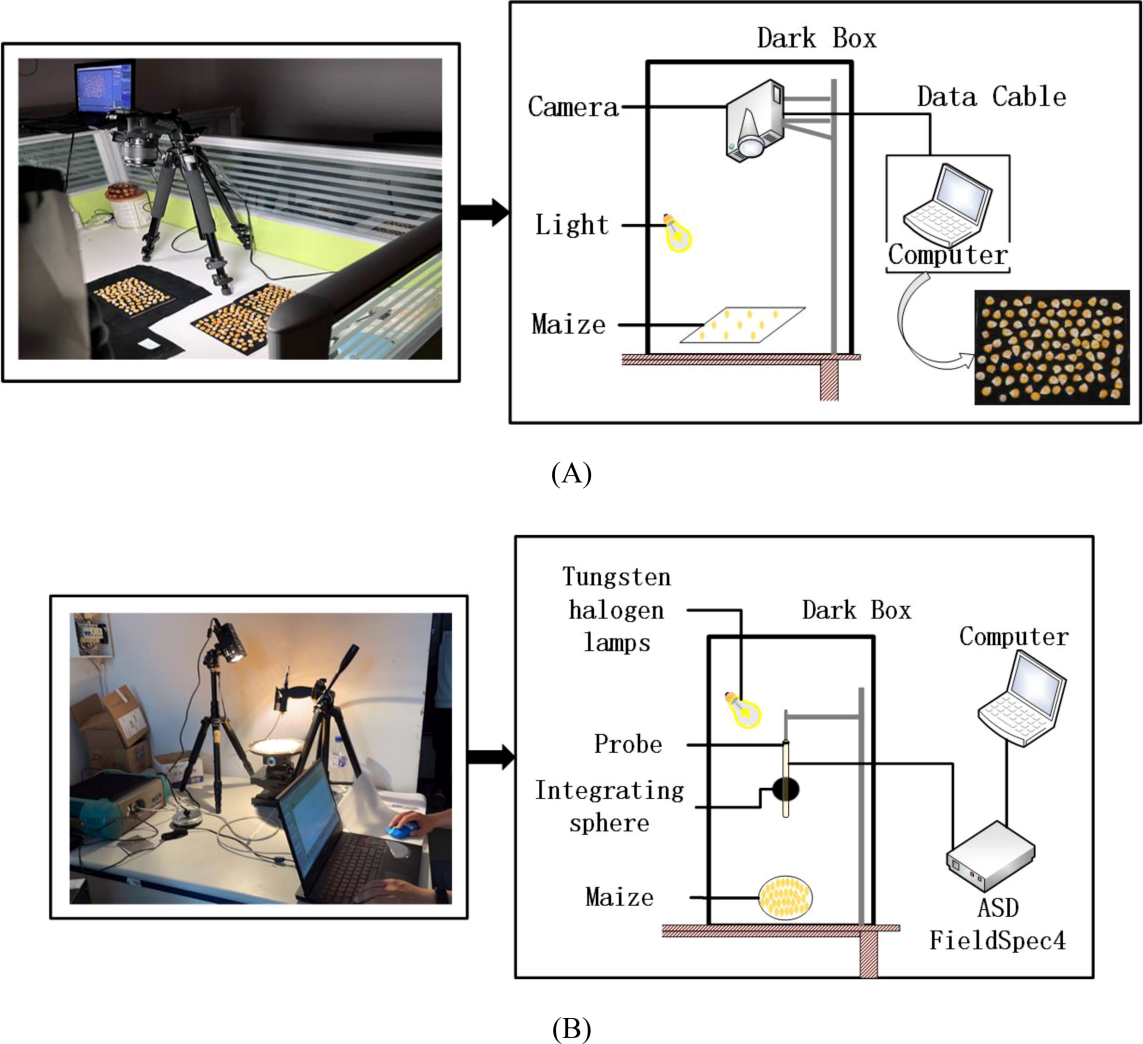
Real image and schematic diagram of maize kernel collection: **(A)** image data; **(B)** hyperspectral data.

Images of the maize kernels were captured by a Canon EOS 1500D camera, and all images were taken under uniform conditions in order to ensure consistency in the data acquisition environment, to avoid interference from external light sources, and to minimize the impact of external factors on image quality. Maize kernels were placed on a black background plate, and the camera was mounted vertically above it with two stabilized LED light sources to provide consistent illumination. The acquisition equipment is shown in **Figure 2A**. The kernels of each variety were divided into groups of 100 and arranged on the black background plate, and a total of 10 sets of images were captured. The resolution of each image was 6000 × 4000 pixels.

#### 2.2.2 Hyperspectral data acquisition

Hyperspectral data from maize kernels were collected with a FieldSpec4 portable spectrometer from ASD, which was used to measure spectral reflectance in the range of 350 to 2500 nm. The distance between the probe and the sample surface was 10 cm, the wavelength accuracy was 0.5 nm, the repeatability was 0.1 nm, the sampling interval was 1 nm, and a 20 W halogen lamp was used as the light source. The schematic diagram of the equipment is shown in **Figure 2B**. Before measurement, the spectra were calibrated by a standard white board, and the average number of measurements was set to 10, with an integration time of 100 ms. 150 maize kernels were randomly selected from each variety for measurement, and the spectrometer was recalibrated before each measurement to ensure data consistency and accuracy. In addition, all measurements were performed in the same laboratory environment in order to minimize the interference of ambient light sources on the measurements. The reliability and reproducibility of the spectral data were ensured through a rigorous experimental design.

### 2.3 Experimental procedure

**Figure 3:**
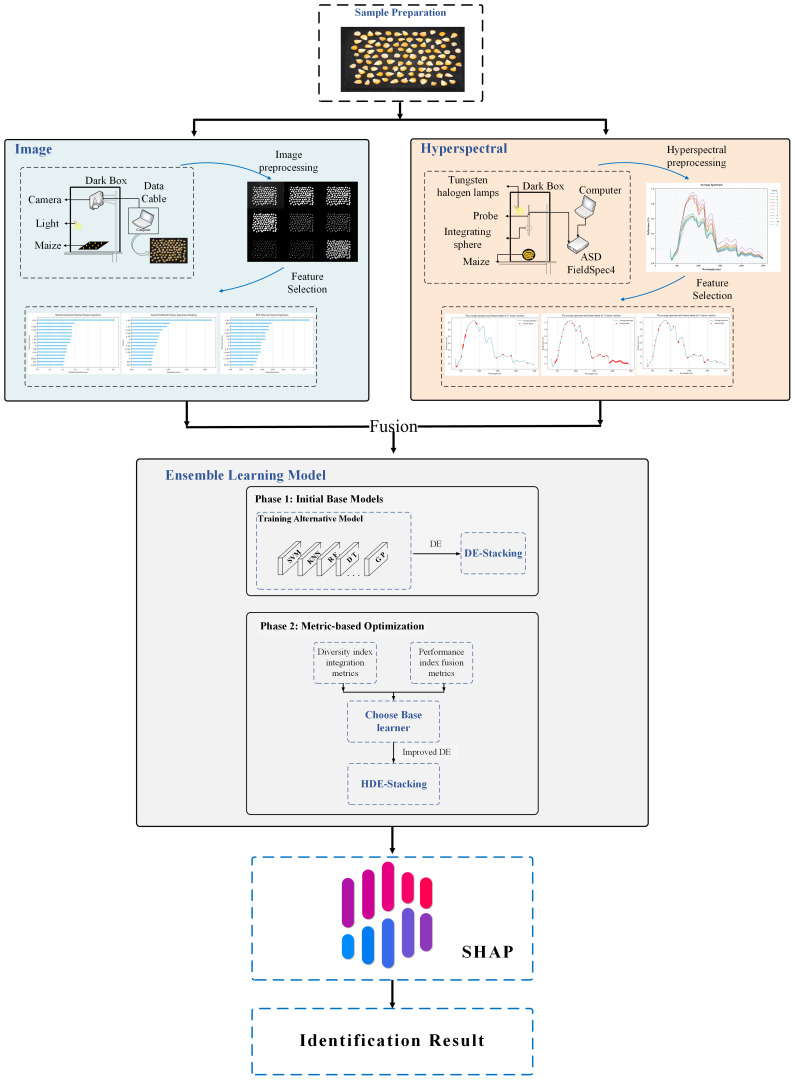
Experimental flow chart.

The computer environment used in this study was as follows: CPU: Intel(R) Xeon(R) Gold 6246R CPU @ 3.40GHz, RAM: 128 GB, GPU: NVIDIA Quadro RTX 8000, 64-bit Windows 10 operating system, Python version 3.8. In order to identify maize kernels, morphological feature data were first extracted from RGB images using the “Machine Vision-based Phenotype Measurement System for Maize Kernels without Reference”, which was developed by the College of Information Technology at Jilin Agricultural University (Software Copyright Registration No.2024SR0703043). Subsequently, the morphological data and hyperspectral data were pre-processed independently, and a subset of features was obtained by a feature selection algorithm. In order to determine the optimal combination pattern, the morphological features of grains, hyperspectral bands and their combinations were used as model inputs. When selecting the base learner for Stacking ensemble learning, the principle of “good but different” was followed, and the differential evolution algorithm was used to optimize the hyperparameters of the ensemble learning model. Then the differential evolution algorithm is improved, and finally the model is interpreted using an interpretable method. The system flowchart is shown in **Figure 3**.

### 2.4 Data pre-processing

It has been shown that seed morphology data can effectively reflect genetic characteristics and are crucial for crop breeding research. In order to retain the relevant information in the seed grain images, the images were first converted to grayscale maps and processed for noise reduction using Gaussian filters. Subsequently, a binarized threshold image is obtained by the Otsu method (Yousefi, 2011; Gómez Ramos et al., 2021), and residual noise is further eliminated using the morphological open operation. Next, the background region is identified by an expansion operation, the foreground region is identified by applying a distance transform, and the unknown region is identified by a subtraction operation. After labeling the foreground region, the watershed algorithm is applied to achieve image segmentation (Longzhe and Enchen, 2011; Seal et al., 2015), and the contour of each maize kernel is extracted using the boundary tracking algorithm. Finally, the minimum outer rectangle was drawn and analyzed morphologically. Geometric, texture and color features of individual kernels were extracted from 1000 kernels of each variety using image processing techniques (Saad et al., 2011; Neuweiler et al., 2020; Khan and AlGhamdi, 2023; Zubair and Alo, 2024), and the un-normalized data may result in some features having too much or too little influence on the model due to the significant differences in the numerical ranges of the different features, thus affecting the performance of the model. Therefore, it is crucial to normalize the data before training the model. This helps ensure that each feature contributes equally to the model.

During hyperspectral data acquisition, phenomena such as diffuse reflection and light scattering on the surface of the sample may cause interference, resulting in significant differences in hyperspectral data for the same type of sample (Cozzolino et al., 2023). This interference not only increases the noise level in the data, but also affects the accuracy of subsequent analysis and model building. Therefore, preprocessing of hyperspectral data is necessary. Hyperspectral preprocessing can effectively reduce the impact of noise on the data, thereby improving the discrimination performance of the model. In this study, the Savitzky-Golay (SG) smoothing technique was used to process hyperspectral data. This technique reduces the impact of random noise by fitting a polynomial within a sliding window, significantly improving the signal-to-noise ratio of the spectral signal while retaining the detailed spectral characteristics. Hyperspectral data processed by SG smoothing more accurately reflects the chemical and physical properties of the sample and significantly enhances the relevance of the data and the discriminative accuracy of the model.

### 2.5 HDE-stacking interpretable integration models

#### 2.5.1 Stacking-based learner selection

In the stacked integration model, the selection of base learners plays a decisive role in the variety identification of maize kernels. The selection of base learners should follow the principle of ‘good but different’ (Sesmero et al., 2021), i.e. each base learner should have excellent discriminatory performance, while at the same time reflecting the differences. Through this approach, the internal features of the maize kernel dataset can be more comprehensively explored in combination with multimodal data, so as to improve the overall performance and generalization ability of the ensemble model. In this paper, a base learner selection strategy that combines diversity and discriminative performance is adopted. However, how to define and evaluate diversity among models is a key research question. Although various diversity metrics have been proposed in the fields of statistics, information theory, and software engineering (Sun et al., 2014; Bian and Chen, 2021), there is no unified standard, which makes diversity metrics somewhat subjective. In addition, the results of a single metric are often not comprehensive and accurate enough. Therefore, this paper proposes a fusion measure of model diversity based on existing research, which fully combines the valid information of different indexes to measure the diversity among models from multiple perspectives for better selection of base learners. The diversity composite index of each candidate model is defined by the combination rule, as shown in (Equation 1).


(1) $$\begin{array}{c} D_{ci_{x}}=\sum_{t=1}^{T}\frac{d_{x,t}}{\sum_{y=1}^{M}d_{y,t}} \end{array}$$

(2) $$\begin{array}{c} d_{x,t}=\{\begin{array}{c} \sum_{y=1}^{M}d_{xy,t},\text{If}\;\text{the}\;\text{metric}\;\text{is}\;\text{bigger},\;\text{the}\;\text{better}\\ \sum_{y=1}^{M}1-d_{xy,t},\text{If}\;\text{the}\;\text{metric}\;\text{is}\;\text{smaller},\;\text{the}\;\text{better} \end{array} \end{array}$$

where $D_{ci_{x}}$ is the diversity composite index of alternative model *x*, *M* is the number of alternative models, *T* is the number of diversity metrics, $d_{xy,t}$ is the difference between model *x* and model *y* under the *T*th diversity measure, and $\;d_{x,t}$ is the sum of the differences between model *x* and other models under the *T*th diversity measure.

The discriminative performance of a model is usually measured by common metrics such as accuracy and recall. Similar to the measure of model diversity, the performance composite of each alternative model can be defined by the combination rule shown in (Equation 3).


(3) $$\begin{array}{c} P_{ci_{x}}=\sum_{s=1}^{S}\frac{a_{x,s}}{\sum_{y=1}^{M}a_{y,s}} \end{array}$$

where, $P_{ci_{x}}$ is the performance composite index of the alternative model *x*, *S* is the number of performance indicators, and $a_{x,s}$ is the *s*th performance indicator value of model *x*.

Combining $D_{ci_{x}}$ and $P_{ci_{x}}$ yields a composite evaluation score for model *x* as shown in (Equation 4).


(4) $$\begin{array}{c} P_{x}=rD_{ci_{x}}+(1-r)P_{ci_{x}},0\leq r\leq 1 \end{array}$$

where r(r=0.5) and 1−r denote the proportion of and in the base learner optimization process, respectively.

In this paper, the dual roles of diversity and accuracy in ensemble learning are emphasized and they are given equal importance. In addition, the alternative models are ranked in descending order based on the comprehensive evaluation scores of the alternative models, and the top K alternative models are taken as the base learners for the Stacking integration models.

#### 2.5.2 Improvement of differential evolutionary algorithms

In order to improve the performance of Stacking ensemble learning models, differential evolutionary algorithms are used to optimize the parameters of the base learner. However, the traditional differential evolutionary algorithm has some limitations in practical applications, which restrict its optimization performance and convergence speed. In this paper, the following two main improvement points are proposed to enhance the performance of the differential evolutionary algorithm in optimizing the Stacking integration learning model.

First, an adaptive control mechanism that dynamically adjusts the mutation factor and recombination rate: the mutation factor (F) and recombination rate (CR) are key parameters in differential evolutionary algorithms, which directly affect the exploration and exploitation capabilities of the algorithms. Fixed variance factor and recombination rate may perform poorly in different optimization stages. In this paper, a dynamic adjustment mechanism is proposed so that these two parameters can be adjusted according to the convergence in the optimization process to improve the adaptability and performance of the algorithm. Let the current iteration number be *t* and the convergence situation be $c_{t}$ , then the dynamic adjustment formula is as follows:


(5) $$\begin{array}{c} F_{t+1}=\{\begin{array}{ll} 0.2+0.5\cdot r, & c_{t}\leq 0.05\\ 0.3+0.5\cdot r,\; & 0.05<c_{t}\leq 0.1\\ 0.5+0.5\cdot r, & c_{t}>0.1 \end{array} \end{array}$$

(6) $$\begin{array}{c} CR_{t+1}=\{\begin{array}{ll} 0.95, & c_{t}\leq 0.05\\ 0.9, & 0.05<c_{t}\leq 0.1\\ 0.7, & c_{t}>\;0.1 \end{array} \end{array}$$

where *r* is a random number in the range [0, 1] used to introduce randomness to avoid premature convergence. With this dynamic adjustment mechanism, the algorithm has strong global search capabilities in the early stages, and in the later stages it focuses more on enhancing local search capabilities, thereby improving the overall optimization effect. Meanwhile, the adaptive control mechanism dynamically adjusts the parameters by monitoring the diversity and convergence of the population. Let the diversity of the current population be $D_{Current}$ ​, and the maximum diversity be $D_{max}$ , then the adjustment formula for the variation factor and recombination rate is:


(7) $$\begin{array}{c} F_{t+1}=F_{base}+\text{Δ}F\cdot (1-\frac{D_{Current}}{D_{max}}) \end{array}$$

(8) $$\begin{array}{c} CR_{t+1}=CR_{base}+\text{Δ}CR\cdot (1-\frac{D_{Current}}{D_{max}}) \end{array}$$

where $F_{base}$ and $CR_{base}$ are the baseline variance factors and recombination rates, and ΔF and ΔCR are the adjustment margins. Through this adaptive control mechanism, the algorithm can automatically adjust the parameters at different optimization stages to improve the overall performance and convergence speed.

Second, the combination of multiple mutation strategies: the mutation strategies used in differential evolution algorithms directly affect the diversity of the population and the algorithm’s global search ability. Traditional differential evolution algorithms usually use a single mutation strategy, such as DE/rand/1/bin or DE/best/1/bin. such a single strategy may not be sufficient to provide enough diversity in some cases, which results in the algorithm easily falling into local optimal solutions. Therefore, this paper proposes to combine multiple variation strategies to enhance the algorithm’s global search and local exploitation capabilities including rand1, best1, current-to-best1 and best2. The variation strategy formulas are as follows:

DE/rand/1/bin strategy:


(9) $$\begin{array}{c} v_{i}^{\left(t+1\right)}=x_{r1}^{\left(t\right)}+F\cdot (x_{r2}^{\left(t\right)}-x_{r3}^{\left(t\right)}) \end{array}$$

where $x_{r1}^{\left(t\right)}$ , $x_{r2}^{\left(t\right)}$ , $x_{r3}^{\left(t\right)}$ are three different individuals chosen at random.

DE/best/1/bin strategy:


(10) $$\begin{array}{c} v_{i}^{\left(t+1\right)}=x_{best}^{\left(t\right)}+F\cdot (x_{r1}^{\left(t\right)}-x_{r2}^{\left(t\right)}) \end{array}$$

where $x_{best}^{\left(t\right)}$ is the current optimal individual, $x_{r1}^{\left(t\right)},x_{r2}^{\left(t\right)}$ are two different individuals chosen at random.

DE/current-to-best/1 strategy:


(11) $$\begin{array}{c} v_{i}^{\left(t+1\right)}=x_{i}^{\left(t\right)}+F\cdot (x_{best}^{\left(t\right)}-x_{i}^{\left(t\right)})+F\cdot (x_{r1}^{\left(t\right)}-x_{r2}^{\left(t\right)}) \end{array}$$

where $x_{i}^{\left(t\right)}$ is the current individual, $x_{best}^{\left(t\right)}$ is the current optimal individual, $x_{r1}^{\left(t\right)},x_{r2}^{\left(t\right)}$ is two different individuals chosen at random.

DE/best/2/bin strategy:


(12) $$\begin{array}{c} v_{i}^{\left(t+1\right)}=x_{best}^{\left(t\right)}+F\cdot (x_{r1}^{\left(t\right)}-x_{r2}^{\left(t\right)})+F\cdot (x_{r3}^{\left(t\right)}-x_{r4}^{\left(t\right)}) \end{array}$$

where $x_{best}^{\left(t\right)}$ is the current optimal individual, and $x_{r1}^{\left(t\right)},x_{r2}^{\left(t\right)},x_{r3}^{\left(t\right)},x_{r4}^{\left(t\right)}$ are four different individuals chosen at random.

The above variant strategies have different advantages at different stages of optimization. The main goal of the initial phase is to explore the search space extensively to find possible high quality solutions, so strategies such as DE/rand/1/bin are suitable to increase the population diversity. The intermediate stage requires finding a balance between exploring new solutions and exploiting existing ones, so strategies such as DE/current-to-best/1 can be used. While in the later stages, the main goal is to fine-tune the exploitation of the better solutions, so strategies such as DE/best/1/bin and DE/best/2/bin are more suitable. By combining multiple variant strategies, the global search and local development ability of the algorithm can be enhanced while maintaining the diversity of the population.

The detailed implementation of the HDE-Stacking integration model is shown in **Table 2**.

**Table 2:** Pseudo-code of Improved DE for Stacking ensemble model optimization.

Input: Population size NP, Number of generations G_max, Initial mutation factor F_base, Initial crossover rate CR_base, Diversity adjustment parameters △F, △CR, Maximum diversity D_max.
1 Initialize population P(t) with NP individuals 2 Initialize base mutation factor F_base and crossover rate CR_base 3 for t = 1 to G_max do 4 Calculate current diversity D_Current of population P(t) 5 6 Adjust F and CR based on convergence indicator c_t: 7 F_(t+1) = dynamic adjustment based on c_t and D_Current 8 CR_(t+1) = dynamic adjustment based on c_t and D_Current 9 for each individual i in P(t) do 10 Select mutation strategy based on optimization stage: 11 v_i^(t+1) = mutation using selected strategy 12 13 Perform crossover to generate trial vector U_i 14 Select the better between U_i and X_i to form new population 15 end for 16 17 Update and monitor the best solution 18 end for 19 20 Return the best solution found

#### 2.5.3 Model interpretability

In machine learning and data science, the interpretability of models has always been a research topic of great concern. For stacking ensemble learning models, their internal mechanisms and decision-making processes are often complex and difficult to understand intuitively. Therefore, the introduction of model interpretability techniques is of great significance for improving the transparency and credibility of models. This paper uses SHAP values to interpret the prediction results of stacking ensemble learning models (Kumar and Geetha, 2022; Huang et al., 2024). The steps are as follows: use diversity metrics and performance metrics to select the base learners of the ensemble learning model, and then use a differential evolution algorithm to optimize the parameters of the Stacking ensemble learning model and train the final model; use the SHAP library to calculate the SHAP values of each feature; visual tools are used to intuitively display the contribution of each feature to the prediction result and the interactions between features; finally, by analyzing the SHAP values, the importance and influence of features in model prediction can be understood, providing guidance for improving and optimizing the model.

## 3 Results

### 3.1 Model evaluation indicators

This paper compares and analyzes the discrimination performance of the model by analyzing the four metrics obtained from the confusion matrix (i.e., accuracy, precision, recall, and F1-Score). At the same time, in order to fully reflect the relationship between models, four common pairwise metrics are used: divergence metric (Dis), q-statistic (Qs), k-statistic (Ks), and correlation coefficient (Cor) (Sun et al., 2014). These paired metrics assess the diversity and relevance of each model from different perspectives, providing richer information for base learner selection. The specific formulas for each metric are detailed in **Table 3**.

**Table 3:** Metrics for model diversity and performance.

Diversity metrics	Formula	Performance metrics	Formula
Dis	$\frac{b+c}{a+b+c+d}$	Accuracy	$\frac{TP+TN}{TP+TN+FP+FN}$
Qs	$\frac{ad-bc}{ad+bc}$	Precision	$\frac{TP}{TP+FP}$
Ks	$\frac{p_{1}-p_{2}}{1-p_{2}}$	Recall	$\frac{TP}{TP+FN}$
Cor	$\frac{ad-bc}{\sqrt{\left(a+b)(a+c)(c+d)(b+d\right)}}$	F1-Score	$\frac{2*P*R}{P+R}$

For models x and y, a and d denote the number of samples correctly and incorrectly identified by the two models, respectively, b denotes the number of samples correctly identified by model x but incorrectly identified by model y, and c denotes the number of samples incorrectly identified by model x but correctly identified by model y. The formula p_1_ = [(a + b)(a + c) + (c + d)(b + d)]/(a + b + c + d)^2^ denotes the probability that the two models realize the chance of agreement, and p_2_ = (a + d)/(a + b + c + d) denotes the probability that the two models realize agreement. True Positive(TP) and False Negative (FN) indicate the number of samples in which the actual positive category was recognized as positive and negative, respectively (Jiang et al., 2023).

### 3.2 Multimodal data fusion

Currently, multimodal data fusion methods are mainly divided into three categories: data layer fusion, feature layer fusion and decision layer fusion (Ilhan et al., 2021). In this study, a feature-level fusion approach was used to integrate image data and spectral data of maize kernels to improve the predictive performance of the model. Image data are used to capture morphological features of maize kernels, while spectral data provide information on the chemical composition and internal structure of maize kernels, and combining these two types of complementary features to form an ensemble feature space allows the model to take advantage of both image and spectral data to improve the accuracy and robustness of identification.

#### 3.2.1 Maize kernel morphological feature extraction

**Figure 4:**
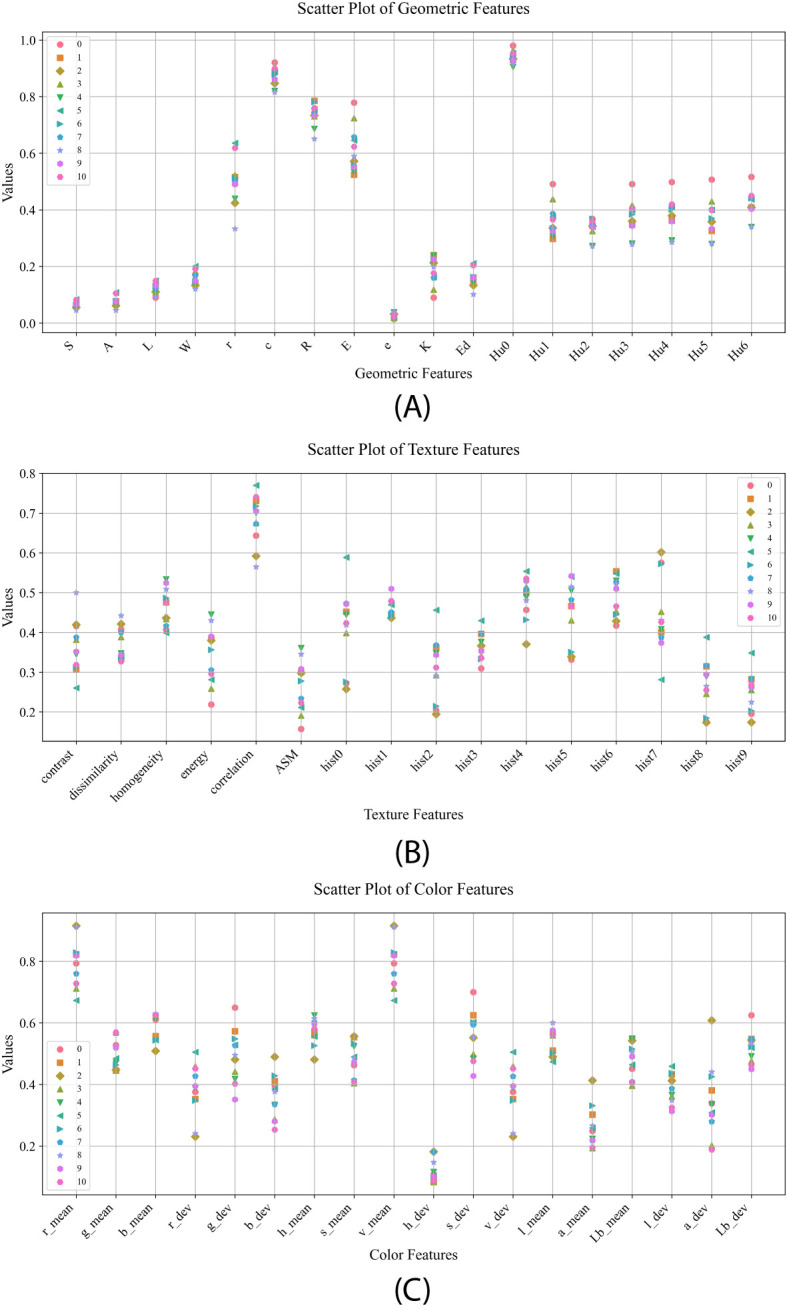
Mean values of morphological features for different seed categories: **(A)** mean values of geometric features; **(B)** mean values of textural features; **(C)** mean values of color features.

In this paper, fifty-two morphological features were extracted from maize kernels, and the normalized mean values in different kernel categories are shown in **Figure 4**. By analyzing the distribution of data points in **Figure 4A**, it is found that the maize kernel shape features are significantly different in different categories, among which the distribution of data points for features ‘E’ and ‘r’ is more scattered, indicating that these features have greater variation among different maize kernels, showing a stronger differentiation ability. The mean values of texture features of different kernel categories are shown in **Figure 4B**, where category JD436 has significant differences from other categories in feature contrast, while category JD50 shows significant discrimination in feature ‘hist0’. The mean values of the color features of different kernel categories are shown in **Figure 4C**, and the distribution of data points for features such as ‘g_mean’, ‘b_mean’, ‘h_mean’, and ‘l_mean’ is more concentrated, indicating that there are less differences in these features among different categories of maize kernels. Comparatively speaking, the features ‘s_dev’, ‘a_dev’ and ‘g_dev’ show significant differences, for example, category JD209 shows obvious distinguishing characteristics on the feature ‘g_dev’, while category JD505 has a high degree of recognition on the feature ‘a_dev’. In summary, variety identification of different types of maize kernels based on morphological features is feasible, and the significant differences in these features provide strong support for identification.

Due to the potential interconnections between the extracted morphological features, not all of them had a significant impact on the construction of the model. Therefore, this study used mutual information (MI), recursive feature elimination (RFE) and significance weighted selection of features (SFM) methods for morphological feature selection.

**Figure 5:**
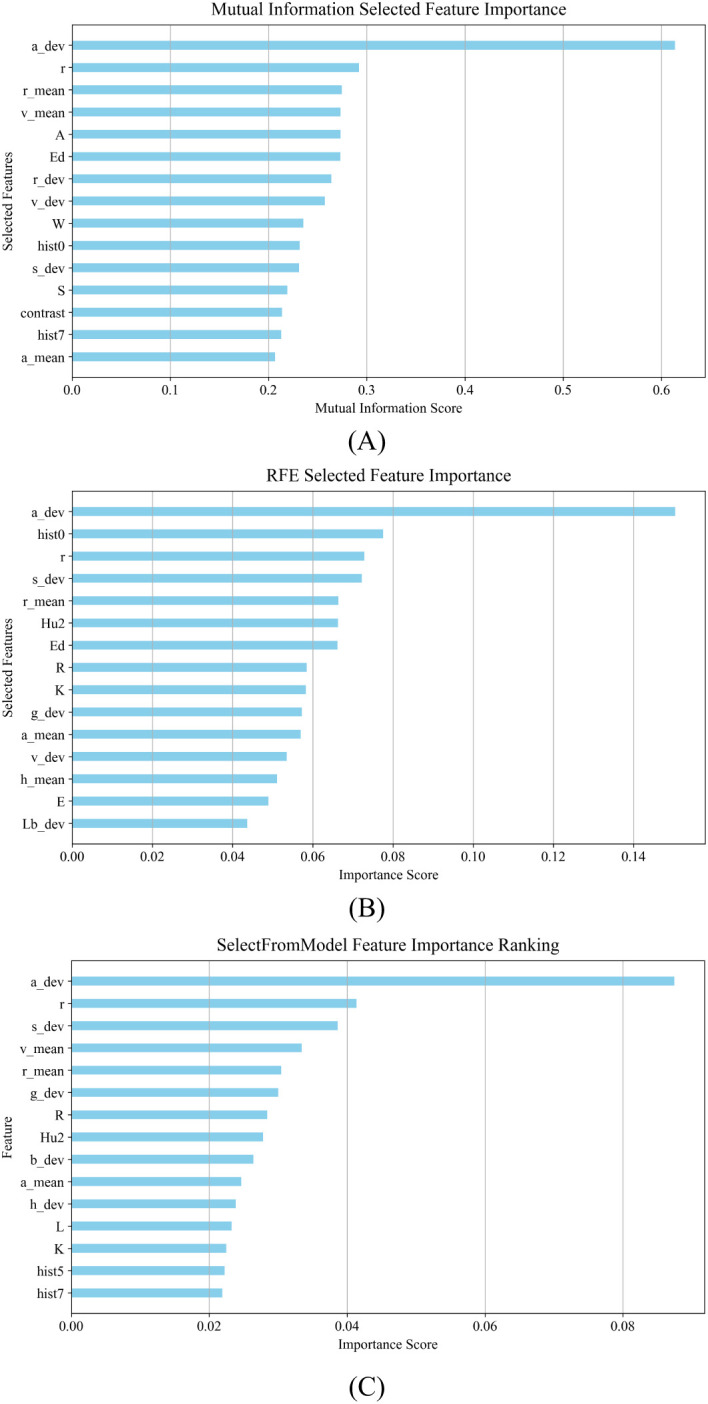
Results of feature selection for morphological characterization of maize kernels: **(A)** results of feature selection using MI; **(B)** results of feature selection using RFE; **(C)** results of feature selection using SFM.

1. MI: The application of MI in classification quantifies the dependency of each feature on the target variable by measuring the amount of mutual information between the features and the target variable to select the set of features that contribute most to the classification result (Kraskov et al., 2004). Mutual information is used to select the top 15 features as shown in **Figure 5A**.
2. RFE: RFE selects a set of features that contribute most to the classification result by training a classification model recursively, evaluating the importance of features, and gradually eliminating the least important features (Darst et al., 2018). RFE is used to select the top 15 most important features, as shown in **Figure 5B**.
3. SFM: SFM is a model-based feature selection method that focuses on features of higher importance as determined by a predefined machine learning model (Raschka, 2018). The top 15 features are selected using a tree-based evaluator, as shown in **Figure 5C**.

**Figure 6:**
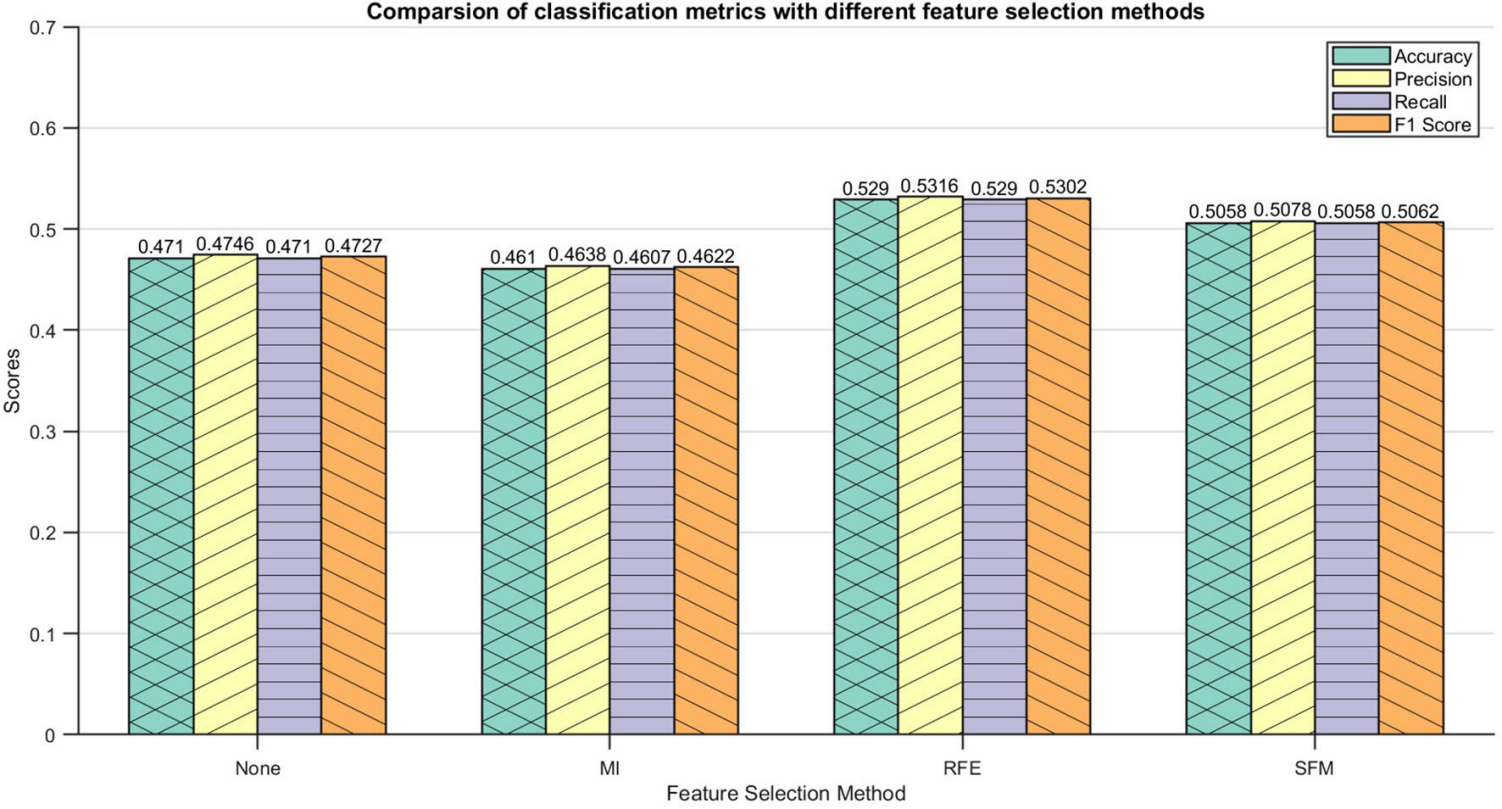
Identification result of DE-Stacking under morphological features.

The normalized morphological features and the feature data after feature selection were input into the DE-Stacking model, and the dataset was divided into a training set and a test set in a ratio of 7:3 for subsequent analysis. The maximum number of iterations of the DE algorithm was 50, and the population size was 20. The Stacking ensemble learning base learner used Logistic Regression (LR), Decision Tree (DT), Support Vector Machine (SVM), k-Nearest Neighbor (KNN) and Gaussian Process (GP). The RFE algorithm outperformed the other two in terms of feature selection. Support Vector Machine, SVM), k Nearest Neighbor, KNN) and Gaussian Process, GP). The RFE algorithm outperformed the other two algorithms in terms of feature selection, as shown in **Figure 6**. Compared with the discrimination results obtained by inputting all morphological features, the results were slightly enhanced by selecting one-third of the features for discrimination. The results show that feature selection can significantly improve the algorithm’s discrimination effect. However, due to the high similarity in appearance of the maize kernels, the model faces challenges in accurately distinguishing between them, resulting in the possibility of some categories being confused. This reflects the limitations of relying solely on morphological features for discrimination, especially when dealing with a large number of categories. Therefore, the introduction of more comprehensive and diverse data to enrich the feature set is crucial to improving discrimination accuracy.

#### 3.2.2 Hyperspectral feature extraction of maize kernels

**Figure 7:**
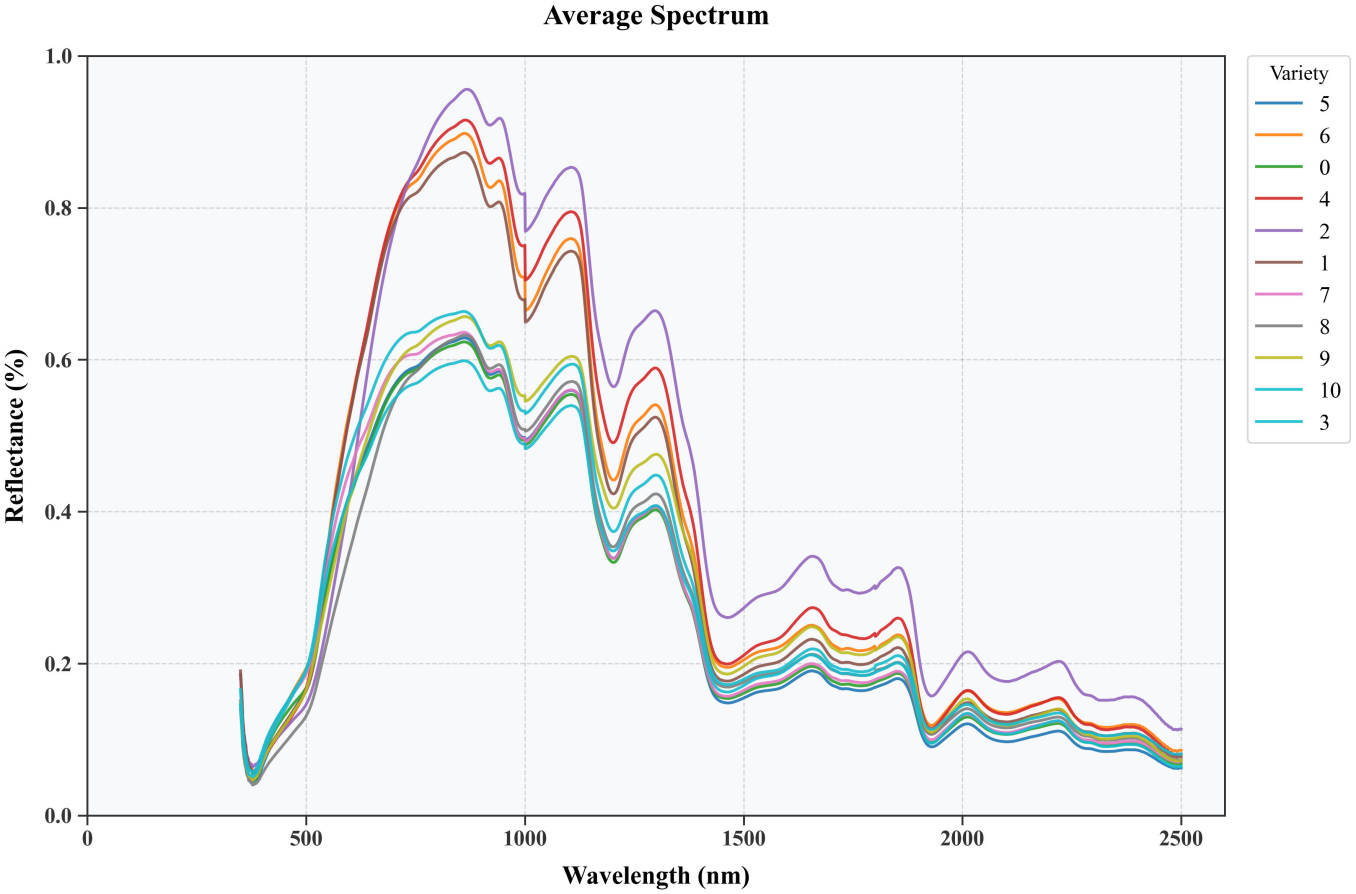
Average reflectance spectra of different maize varieties after pre-treatment with an SG filter.

The spectral curve is processed using the SG smoothing algorithm, which effectively reduces the impact of noise. Maize kernels have a wide distribution in the near-infrared spectral region, but the overall trend of the spectral curves of all varieties is similar, with obvious peaks near 863 nm, 1105 nm, 1295 nm, 1680 nm and 2015 nm, and obvious valleys near 980 nm, 1175 nm, 1450 nm, 1780 nm and 1915 nm, as shown in **Figure 7**. These spectral features reflect the differences in protein, fat, and carbohydrate content between different maize varieties, which are due to the different absorption strengths of the C-H, N-H, and O-H groups in organic components in these spectral ranges. Interesting correlations exist between spectral and morphological characteristics. For example, varieties with higher ‘E’ values tended to show stronger absorption in the 910 nm band, suggesting a possible relationship between kernel shape and protein-water interactions. This correlation may be due to the effect of kernel shape on the internal water distribution pattern. The absorption peaks observed near 863 nm, 1105 nm and 1295 nm showed different intensities in varieties with different surface texture characteristics. Varieties with higher logarithmic values in the texture analysis usually showed stronger absorption at these wavelengths, suggesting a potential link between surface structure and internal biochemical composition. The relationship between color features and spectral features is particularly evident in the visible range (400-700 nm). Species with higher color standard deviations (‘s_dev’, ‘a_dev’) showed more complex spectral patterns in this range, suggesting that the heterogeneity of the surface color may reflect potential changes in pigment distribution and composition. Therefore, these differences form the basis for the use of hyperspectral data for seed discrimination in agricultural applications.

Due to the presence of co-linear spectral bands in hyperspectral data, there is significant redundancy, which leads to an increase in model training time and a decrease in accuracy. Therefore, it is necessary to select characteristic bands before inputting data into the model to reduce the impact of redundant bands on the model. This study uses the sequential projection algorithm (SPA), the competitive adaptive reweighted sampling algorithm (CARS), and the bootstrap soft shrinkage algorithm (BOSS) for morphological feature selection.

**Figure 8:**
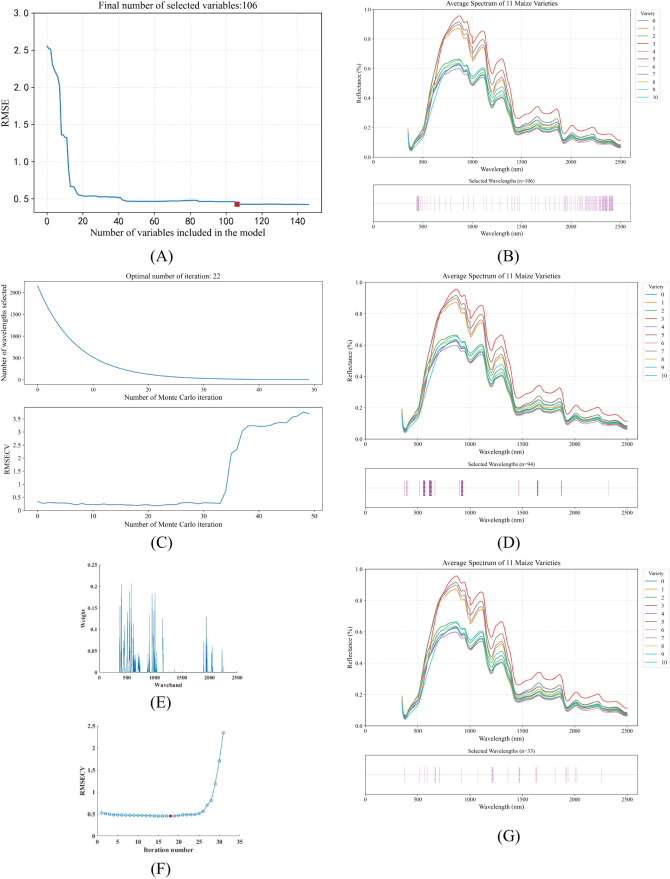
Visualization of feature wavelength selection results using three different algorithms. **(A)** RMSE change curve with increasing number of feature wavelengths selected by SPA algorithm **(B)** Index distribution of 106 feature wavelengths identified by SPA algorithm **(C)** RMSECV curve with increasing number of feature wavelengths selected by CARS algorithm **(D)** Index distribution of 94 feature wavelengths identified by CARS algorithm **(E)** Visualization of wavelength band weights determined by BOSS algorithm **(F)** RMSECV change curve with increasing number of iterations in BOSS algorithm **(G)** Index distribution of 33 feature wavelengths identified by BOSS algorithm.

1. SPA: SPA is a forward feature selection method that uses vector projection analysis. By projecting wavelengths onto other wavelengths and comparing the magnitudes of the projected vectors, the wavelength with the largest projected vector is selected as the candidate wavelength, and then the final feature wavelengths are selected based on the correction model (Soares et al., 2013). SPA selects a combination of variables with minimal redundant information and minimal collinearity. Therefore, the characteristic wavelengths were selected using SPA from 2151 bands between 350 nm and 2500 nm. The process of selecting the spectral bands is shown in **Figures 8A, B**.
2. CARS: The basic idea of CARS is to adaptively adjust the selection probability of each band through Monte Carlo sampling and an exponential decay function, and finally select the optimal band combination that contributes the most to modeling performance (Li et al., 2009). As the number of runs increases, the selected characteristic wavelengths gradually decrease. After twenty-two runs, an ideal feature subset is obtained, as shown in **Figures 8C, D**.
3. BOSS: The BOSS algorithm is a variable selection method proposed by Baichuan Deng and others, which is specifically used for near-infrared spectroscopy data analysis. Through Bootstrap sampling and soft shrinkage techniques, the feature variables that contribute most to the model are evaluated and selected recursively, thereby effectively reducing the data dimension, reducing model complexity and multiple collinearity, and improving model stability and prediction performance (Al-Kaf et al., 2020). As shown in **Figures 8E–G**, the RMSECV reaches a minimum at the 18th iteration, at which point the BOSS algorithm selects 33 eigenbands.

**Figure 8** show the selected spectral characteristic wavelength groups for each maize kernel variety using the SPA, CARS and BOSS algorithms, respectively. The SPA algorithm reduces the original 2151 bands to 106, CARS reduces the characteristic wavelengths to 94, and BOSS reduces the characteristic wavelengths to 33, greatly reducing the input data for the model.

**Figure 9:**
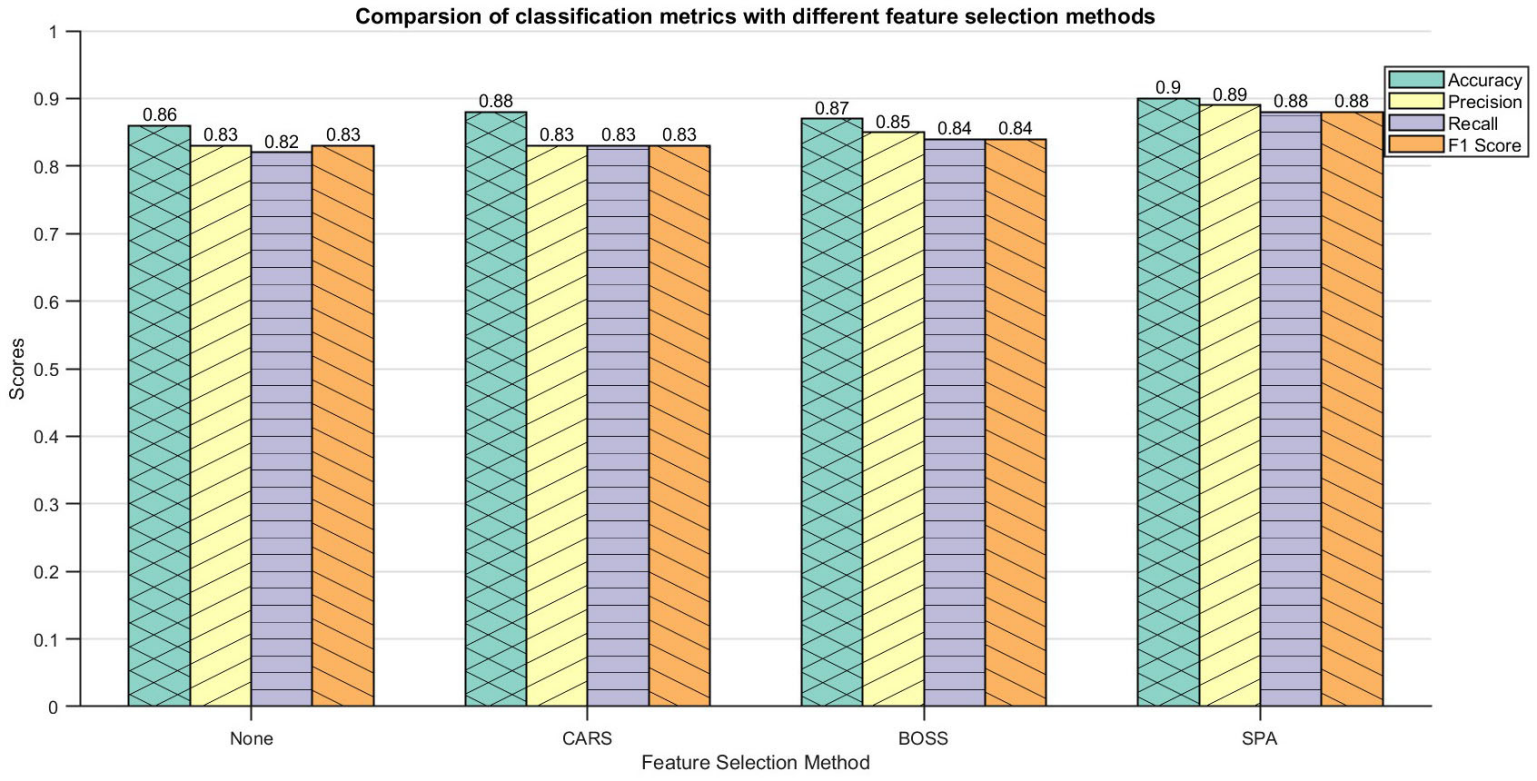
Identification result of DE-Stacking under spectral feature.

The training and test sets of the DE-Stacking-based hyperspectral data discrimination model for maize kernels were randomly selected from each category at a ratio of 7:3 from a pool of 150 samples. The discrimination results are shown in **Figure 9**.

#### 3.2.3 Data fusion

**Figure 10:**
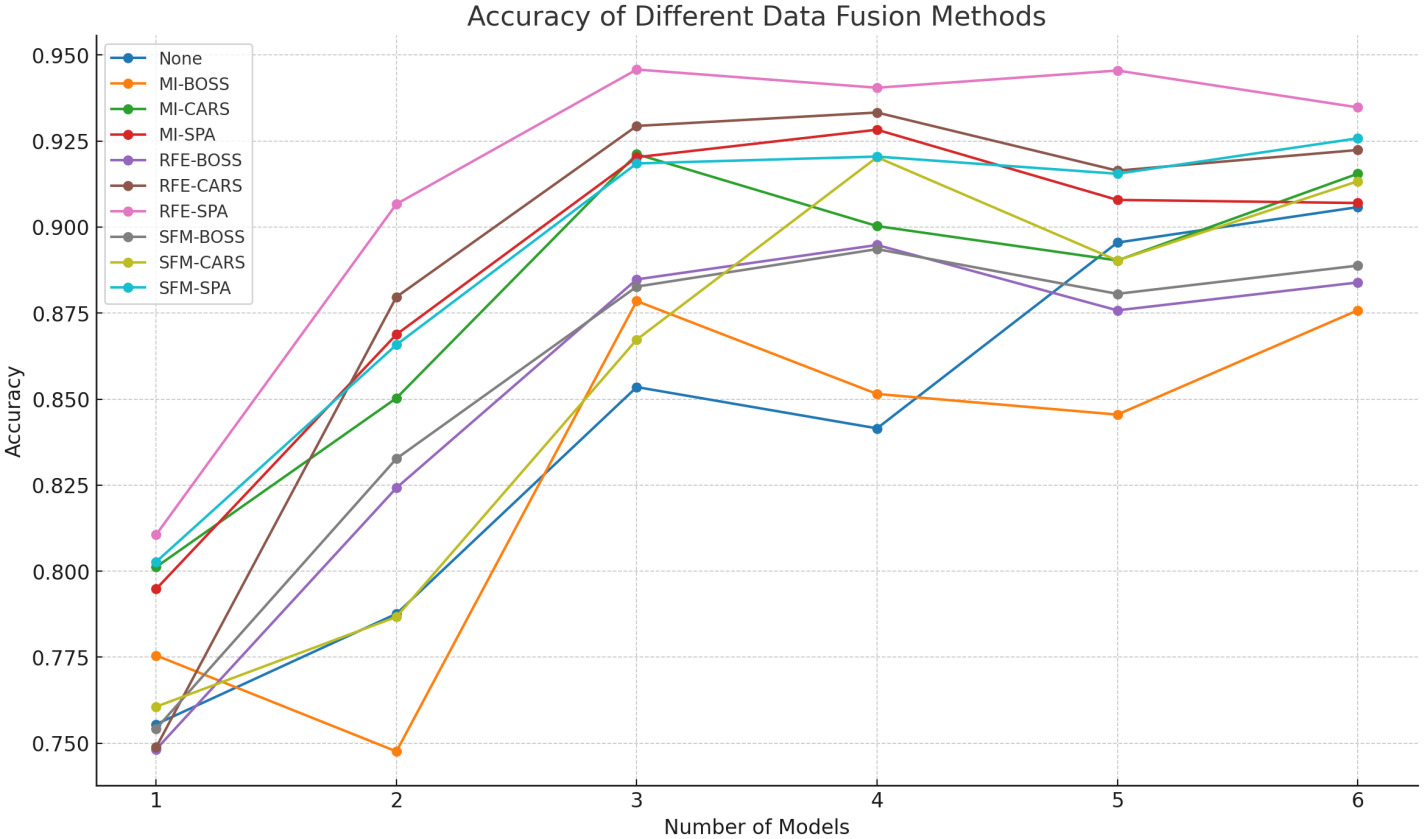
Comparison of the identification results using the DE-Stacking model after data fusion.

It is known that spectral bands can reflect the selectivity of different varieties of maize kernels in terms of reflection, absorption and transmission of incident radiation. In addition, the morphological characteristics of the kernels can provide insight into their surface properties, as well as structural and organizational changes that are not visible to the naked eye. Therefore, fusing morphological data with spectral data can provide richer feature information for model training. To construct the maize variety identification model, we combined morphological features selected by MI, RFE and SFM with spectral features selected by different methods (33 wavelengths from BOSS, 106 from SPA, and 94 from CARS). The results in **Figure 10** show that combining RFE-selected morphological features with SPA-selected spectral features achieved the highest accuracy among all nine feature combinations when input to the DE-Stacking model. This feature-level fusion improved accuracy by 3.1% compared to using all morphological and spectral features, demonstrating that selective information fusion can enhance both model accuracy and stability while reducing misclassification errors.

### 3.3 Alternative model evaluation

**Figure 11:**
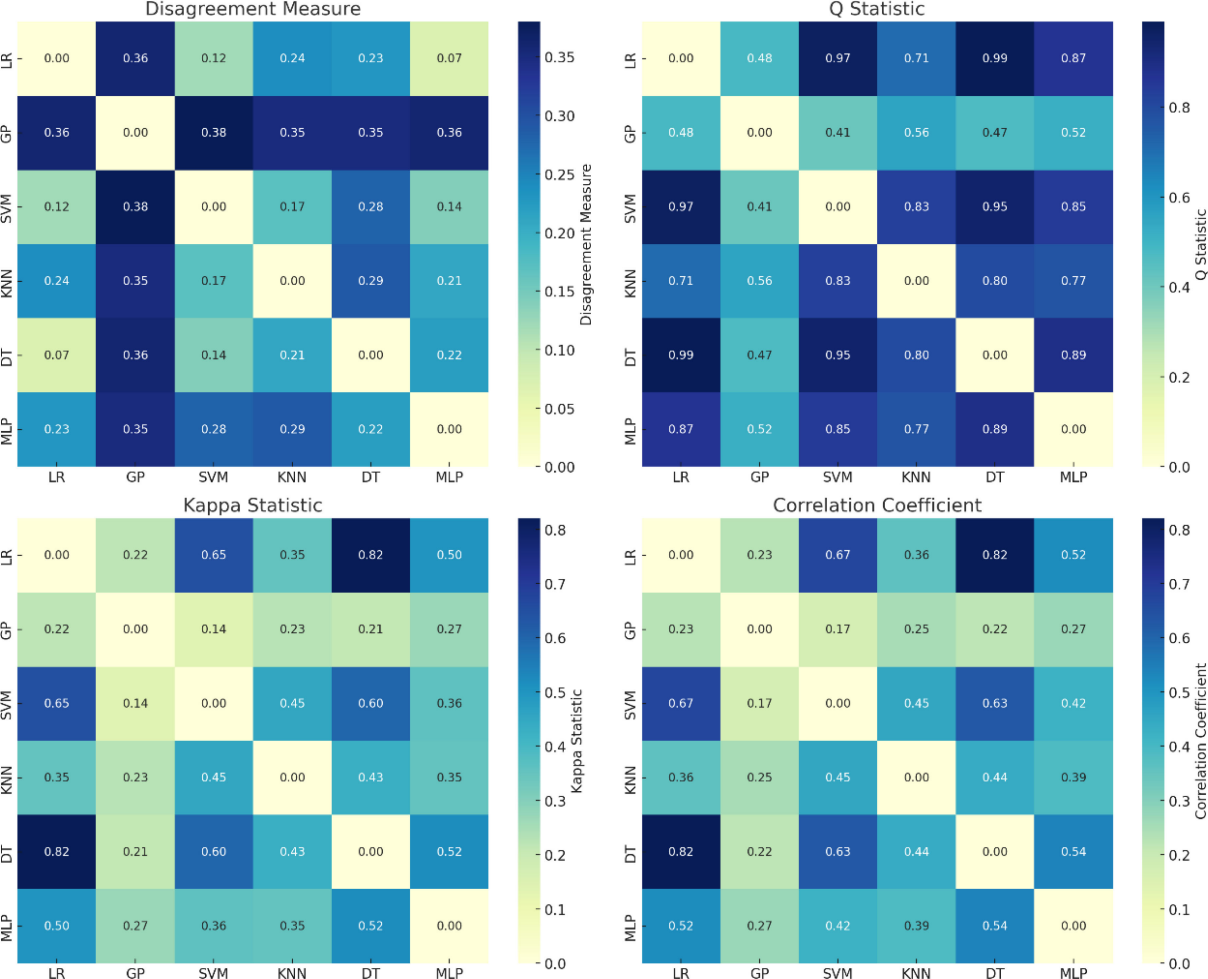
Diversity measures for alternative models.

Using six single models as base learners has long processing times and high complexity, so the number of base models needs to be reduced while maintaining the accuracy. This study is based on a dataset with RFE-SPA feature fusion, and the discrimination results of each alternative model on the test set are compared and analyzed using five-fold cross-validation. The performance and diversity metrics in **Table 3** are used to evaluate the models, and the discrimination performance and difference distribution of the six candidate models are shown in **Table 3** and **Figure 11**.

**Table 4** shows that the DT model performed the worst in individual prediction, with an accuracy, precision, recall, and F1-Score of less than 65%, while the performance indicators of the remaining five models were all above 68%. For the small data set of maize grain varieties studied in this paper, the MLP model performed best in the four evaluation metrics, and the SVM model has a unique advantage in processing high-dimensional small sample data. The LR model can effectively capture the linear relationships in the data by mapping the linear combination of input features to the probability space, and also shows better discrimination ability.

**Table 4:** Discriminatory performance of alternative models.

Models	Accuracy (%)	Precision (%)	Recall (%)	F1-Score (%)
LR	73.33	73.12	74.5	73.81
DT	61.52	63.15	61.9	61.52
MLP	82.12	83.65	82.43	83.03
KNN	70.00	70.8	69.75	70.27
GP	73.03	74.56	72.98	73.76
SVM	72.73	72.45	73.33	72.89

**Figure 11** shows the heat maps of the Disagreement Measure (Dis), Q Statistic (Qs), Kappa Statistic (Ks) and Correlation Coefficient (Cor) respectively. The darker the color, the greater the diversity between the models, and vice versa. Although the results obtained using different diversity measures are different, they generally reflect the same trend in model diversity. In addition, since the training mechanisms of SVM, KNN, GP and LR differ greatly from those of other learners, the diversity indicators Dis, Qs, Ks and Cor for these learners also show significant differences.

The choice of the base learner needs to take into account both the discriminative ability of the model and the differences between the models. Therefore, the diversity and performance indices of each model were calculated and ranked according to Eqs. (1), (3), and (4), respectively, as shown in **Table 5**. It can be seen that under the influence of the model diversity index, the MLP model with the best discriminative performance only ranked 3rd, while the DT model with the worst discriminative performance ranked 2nd in the overall ranking.

**Table 5:** Metrics and rankings for alternative models.

Models	Diversity composite index	Performance composite index	Comprehensive evaluation score	Comprehensive ranking
LR	0.7707	0.3472	0.5589	1
DT	0.7297	0.2864	0.5580	2
SVM	0.6889	0.3432	0.5160	4
KNN	0.5788	0.3182	0.4485	5
GP	0.4819	0.3364	0.4091	6
MLP	0.6710	0.3773	0.5241	3

**Figure 12:**
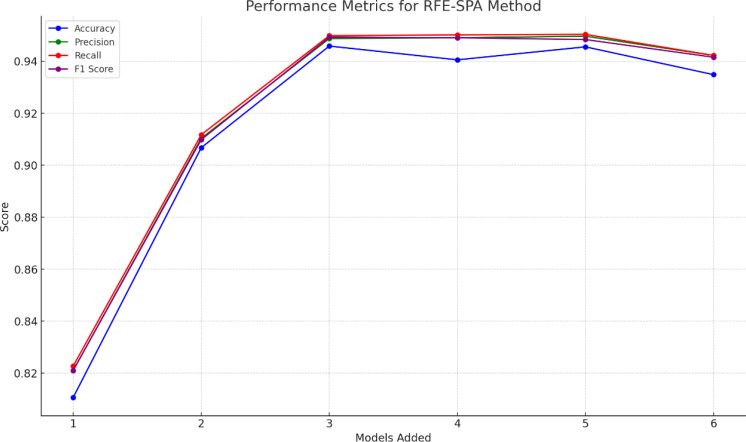
Performance comparison of ensemble models with different numbers of base learners.

To obtain the best ensemble model and verify the rationality of the base learner selection method in this paper, experiments were conducted using the RFE-SPA fusion data. The model with a number of base learners of 1 uses the base learner with the highest overall ranking in **Table 5**, the model with a number of 2 uses the top two, and the model with a number of 3-6 uses the top three, and so on, as shown in **Figure 12**.

**Table 5** combined with **Figure 12** shows that as the number of base learners increases, the overall performance of the ensemble model tends to first increase and then decrease. This is because not all base learners can provide gain. When there is a large performance difference between base learners, the ensemble model often relies on the better performing learners, while the contribution of the worse performing learners may be ignored or even have a negative impact. As shown in **Figure 12**, the addition of SVM reduces the performance of the ensemble model with 3 base learners. If the newly added base learner is similar to the existing learners, it may produce similar errors, resulting in no improvement in the performance of the ensemble model, or even worse than a single excellent base learner. The addition of GP and KNN also resulted in a decrease in performance, as they are less diverse and do not have a significant advantage in accuracy, but instead increase the computational complexity of the model.

Ensemble model 1 adopts the base learner selection strategy proposed in Section 3.2 and is the model with the best performance in **Figure 12**. Ensemble models 2 and 3 only consider the differences between models and select models with higher diversity indices in **Table 5** as base learners. Ensemble models 4 and 5 only consider the identification performance of models and select models with higher accuracy as base learners. Ensemble models 6 and 7 randomly select models in **Table 5** as base learners, as shown in **Table 6**. Interestingly, some ensemble models with randomly selected base learners performed well, possibly because the random selection strategy introduced more diversity. In addition, although the ensemble methods that only consider the diversity or accuracy of the base learners can improve the performance of the ensemble model to a certain extent, the improvement is limited. On the one hand, these ensemble methods may include base learners with lower performance, which is difficult to significantly improve the overall performance of the ensemble model; on the other hand, the accuracy of the base learners is similar, which fails to provide sufficient diverse information for the model and limits the improvement of performance. After analyzing **Table 6**, this paper finally selects DT, LR, and MLP as the base learners for the DE-Stacking ensemble model. This combination balances the number of base learners while taking into account accuracy and diversity, effectively improving the performance and stability of the model and reducing the error in the identification of maize kernel varieties.

**Table 6:** Performance comparison of ensemble models with different base learner combinations.

Ensemble Models	Combinations of base learners	Accuracy (%)	Precision (%)	Recall (%)	F1-Score (%)
1	LR+DT+MLP	94.58	94.87	94.98	94.92
2	LR+SVM+MLP	91.79	92.05	91.96	92.00
3	LR+SVM+DT	88.96	89.45	89.39	89.42
4	LR+SVM+GP	86.08	86.76	86.68	86.65
5	SVM+GP+KNN	82.57	82.83	82.75	82.69
6	DT+KNN+GP	83.01	83.4	83.49	83.47
7	KNN+GP+MLP+DT	91.10	91.58	91.47	91.51

### 3.4 Comparison of models

In order to further validate the discriminatory performance of the ensemble model, the test set of maize variety fusion data was predicted and analyzed using MLP, RF represented by Bagging, XGBoost represented by Boosting, and DE-Stacking ensemble model, and the discriminatory results are shown in **Table 7**. The DE-Stacking ensemble model of accuracy, precision, recall and F1-Score reached 94.58%, 94.87%, 94.98% and 94.92%, respectively, which is 4.7 percentage points higher than the XGBoost model, which is the best performer among the single prediction models, and 18.25, 17.87, 18.59 and 18.37 percentage points higher than the average of all models, respectively. The DE-Stacking integration model proposed in this paper shows better overall discrimination performance. This is because a single model can only classify the categories of maize kernels from a specific perspective, and the boundaries of maize kernel categories often have large ambiguities and uncertainties, which makes it difficult for a single model to achieve comprehensive and accurate discriminative modeling. The DE-Stacking model reduces the risk of falling into a local optimal solution during the model training process by integrating multiple base learners with large differences, and can effectively overcome the inherent limitations of a single model in the hypothesis space.

**Table 7:** Performance comparison of different models.

Models	Accuracy (%)	Precision (%)	Recall (%)	F1-Score (%)
MLP	82.12	83.65	82.43	83.03
RF	88.06	88.68	87.55	87.93
XGBoost	89.88	89.63	88.72	89.17
DE-Stacking	94.58	94.87	94.98	94.92

Since the DE algorithm may fall into a local optimum, the DE algorithm is improved using the improvement method in Section 2.5.2 and compared with other optimization methods. The results are shown in **Table 8**. The HDE-Stacking model has the highest accuracy rate of 97.78% compared to the other four models. Compared with the unimproved DE-Stacking model, the accuracy rate has increased by 3.21%, and compared with ABC-Stacking and PSO-Stacking, HDE-Stacking also achieved a better accuracy rate. In terms of precision, the HDE-Stacking model improved by 3.02% compared with the DE-Stacking model on the data set. In terms of recall, the HDE-Stacking model also improved compared with other models on the dataset.

**Table 8:** Comparison of the results of different optimization algorithms.

Models	Accuracy (%)	Precision (%)	Recall (%)	F1-Score (%)
HDE-Stacking	97.78	97.89	97.93	97.91
DE-Stacking	94.58	94.87	94.98	94.92
GWO-Stacking	93.83	94.34	93.86	94.10
ABC-Stacking	91.04	92.87	90.51	91.67
PSO-Stacking	93.34	94.55	93.53	94.04

**Table 9** shows the comparative performance of HDE-Stacking and DE-Stacking models using different feature combinations. Using all morphological features alone, the HDE-Stacking model achieved 55.15% accuracy, while using all hyperspectral features alone reached 92.12% accuracy. The combination of RFE selected morphological features and SPA selected spectral features achieved the highest accuracy of 97.78%. This shows that information fusion effectively improves the accuracy and stability of discrimination while reducing misidentification.

**Table 9:** HDE-Stacking and DE-Stacking models using different feature combinations.

Models	Dataset	Accuracy (%)	Precision (%)	Recall (%)	F1-Score (%)
HDE-Stacking (LR+DT+MLP)	ALL Morphological features	55.15	54.08	55.15	54.40
	ALL Hyperspectral features	92.12	92.23	92.12	92.00
	RFE-SPA features	97.78	97.89	97.93	97.91
DE-Stacking (LR+DT+MLP)	ALL Morphological features	48.09	48.45	48.09	48.26
	ALL Hyperspectral features	88.29	85.04	84.29	85.62
	RFE-SPA features	94.58	94.87	94.98	94.92

### 3.5 Model interpretation

SHAP (Shapley Additive Explanations) is a game-theory-based explanation method for interpreting the predictions of machine learning models. The SHAP values are calculated separately for each base learner. The SHAP values quantify the influence of each feature on the prediction result by calculating the marginal contribution of the feature in different combinations. For different types of base learners, the corresponding SHAP interpreters are used for calculation. After training the stacked model, the weights of each base learner are obtained from the meta learner. These weights reflect the relative importance of each base learner in the final prediction result. Subsequently, the SHAP values of each base learner are multiplied by their corresponding weights, and these weighted SHAP values are then summed to obtain the SHAP values of the stacked model. This process allows the contribution of each base learner to be reflected in the final explanation. Finally, the weighted average of the SHAP values is used to generate relevant explanation charts, through which the decision-making process of the stacked model can be visually understood.

#### 3.5.1 SHAP summary plot

SHAP Summary Plot is used to show the impact and importance of each feature on the model’s prediction results, helping to visually understand the decision-making process of complex models. The horizontal axis represents the SHAP value, which is the magnitude and direction of the impact of each feature on the model output. A positive SHAP value indicates a factor’s positive impact on maize variety identification. In contrast, negative values indicate an attenuating effect. The vertical axis lists all the features used in the model, including hyperspectral and morphological data. These features are arranged in order of importance from top to bottom, with the most important features at the top, and the color indicates the magnitude of the feature value, with red indicating a high feature value and blue indicating a low feature value.

**Figure 13:**
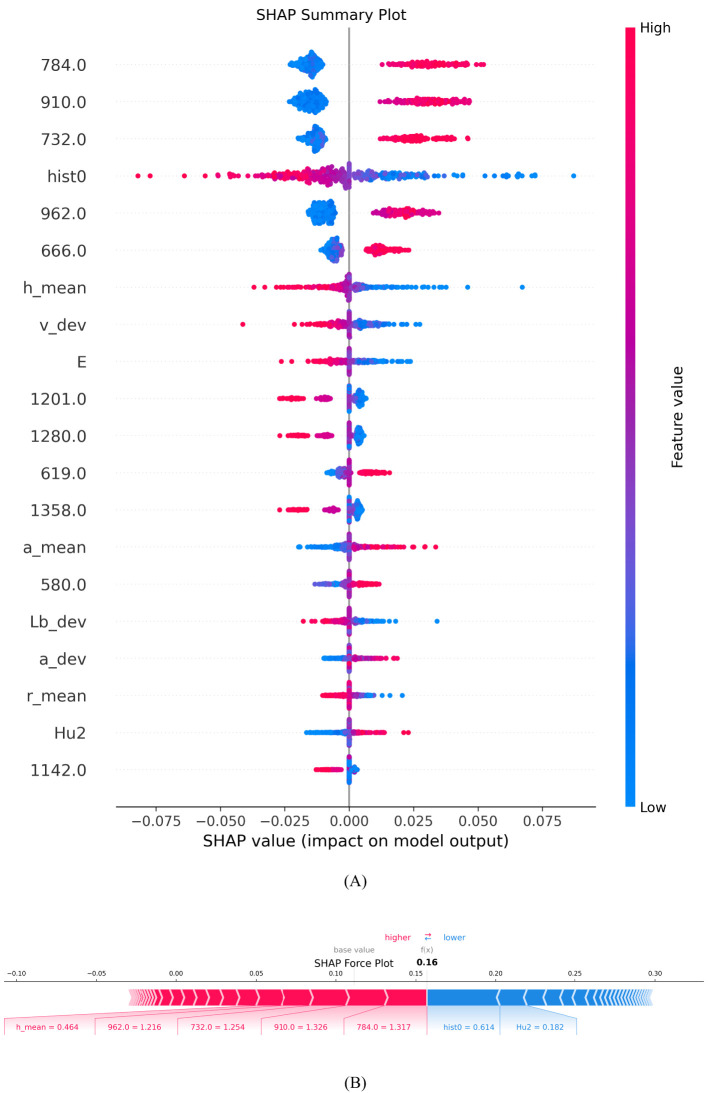
Characteristic contribution explanation diagram. **(A)** SHAP Summary Plot; **(B)** SHAP Force Plot.

As can be seen in **Figure 13A**, the red and blue points of the morphological feature ‘hist0’ are almost evenly distributed near the zero point of the SHAP value, indicating that this feature has a relatively neutral impact on model prediction, with neither a significant positive nor a significant negative impact, indicating its weak predictive ability. The red dots of ‘h_mean’ are concentrated in the negative direction of the SHAP value, indicating that a higher feature value will lead to a lower prediction result; while the red dots of ‘a_mean’ are concentrated in the positive direction, indicating that a high feature value will increase the prediction result. The influence of the “E” feature on the model is small, and it is mainly negative, indicating that a high value may slightly reduce the probability of the model predicting a certain category, but the overall influence is not significant. Nevertheless, the synergistic effect of this feature still needs to be considered in the comprehensive morphological feature analysis.

SHAP analysis showed that multiple spectral bands (784 nm, 910 nm, 732 nm, 962 nm and 666 nm) had significant positive effects on the identification of varieties. This is highly consistent with the known spectral characteristics of corn grains: The 666 nm band falls within the red light region (around 660 nm), where chlorophyll has strong absorption. Its positive contribution suggests that different varieties may have distinct chlorophyll content patterns. The 732 nm band lies in the critical transition zone (691-730 nm) where different maize varieties show significant variation in nitrogen content-related spectral reflectance. This explains why this band contributes positively to variety discrimination. The 784 nm band is close to the 790 nm absorption peak associated with O-H, N-H, and C-H groups in proteins and water, providing important biochemical information for variety differentiation. The 910 nm and 962 nm bands are near the 1000 nm region, where the second overtone of O-H stretching from water-protein interactions occurs. Their positive contributions indicate that varieties differ in their protein and moisture compositions. Conversely, the bands at 1201 nm, 1280 nm, and 1358 nm showed different impacts in variety discrimination. These wavelengths are primarily associated with carbohydrate content (around 1200 nm) and protein C-H stretch first overtone (around 1300 nm). Their varying contributions to variety discrimination may reflect the complex biochemical differences among maize varieties in terms of their carbohydrate and protein compositions. This finding provides an important basis for optimizing band selection. Morphological features show a weaker contribution than spectral features. This indicates that despite the visible morphological differences between varieties, the internal biochemical composition reflected in the spectral data provides more reliable discriminant information for variety recognition. These insights can help agricultural experts optimize grain identification procedures and further advance precision agriculture technologies based on spectral and morphological data.

#### 3.5.2 SHAP force plot

Force Plot is used to interpret the model prediction results for individual samples, showing the impact of each feature on the final prediction value. The length and direction of the arrow are used to indicate the size and direction of the contribution of different features to the model prediction. Red arrows indicate a positive contribution, meaning that these features increase the probability of the sample being identified as the current category; blue arrows indicate a negative contribution, meaning that these features reduce the probability of the sample being identified as the current category. The length of the arrow reflects the contribution of each feature to the prediction result.

**Figure 13B** shows that the Base Value is about 0.11, which indicates the average predicted output value of the model when there is no feature information. The final predicted value f(x) is 0.16, which indicates that the influence of specific features has slightly improved the model’s predicted value compared to the Base Value. The red part on the left side of the figure represents the features that contribute positively to the model output, such as h_mean = 0.464, 962.0 = 1.216 and 910.0 = 1.326, which together push up the predicted value; the blue part on the right side indicates the features that features that have a negative impact on the predicted value, such as hist0 = 0.614 and Hu2 = 0.182. Although they offset the impact of some positive features, they are not enough to completely offset the boosting effect of the red features, so the final predicted value is slightly higher than the baseline value, reflecting the cumulative effect of the positive features. This analysis helps to gain a deeper understanding of the model’s decision-making process in maize kernel variety identification, especially the specific contributions of different morphological and spectral features to the identification results, providing a scientific basis for kernel identification in precision agriculture.

## 4 Discussion

This study selected 11 representative maize varieties from Northeast China, including widely cultivated mainstream varieties such as JiDan27 and JiDan50. Although these varieties are difficult to distinguish by visual inspection, multimodal data analysis revealed significant differences in their morphological and spectral characteristics. Analysis showed that among the fifty-two extracted morphological features, features such as ‘a_mean’ and ‘hist0’ made important contributions to variety discrimination. Particularly in terms of texture features, variety JiDan436 showed significant discrimination in contrast features, while variety JiDan50 demonstrated distinctive characteristics in ‘hist0’ features. Furthermore, in spectral feature analysis, although the overall trends of spectral curves were similar across varieties, they exhibited different reflection intensities at key bands such as 784 nm and 910 nm, reflecting differences in internal kernel compositions. Through feature-level fusion strategy, this study successfully integrated these subtle but crucial morphological and spectral differences to achieve accurate identification of visually similar varieties. However, compared to Huang et al. (2016b), this study has room for improvement in sample diversity. By combining the ISVDD algorithm for classifying maize seeds from different years, they improved classification accuracy from 84.1% to 94.4% after increasing the training set by 11.0% to 12.8%. This indicates that model updating strategies can significantly enhance classification performance when dealing with maize variety identification across different years. Therefore, future research could consider: (1) expanding the sample range to include varieties from other producing areas; (2) introducing model updating mechanisms while maintaining the advantages of regionally representative varieties; (3) conducting multi-year studies to analyze the impact of inter-annual climate variations on morphological and spectral characteristics of maize kernels.

While the proposed HDE-Stacking ensemble model achieved remarkable results in this example verification, the performance of some ensemble model combinations based on selection strategies also gradually improved. Due to the large number of alternative models and random combinations, it is difficult to fully verify all combinations due to time and computational cost constraints. This aligns with the findings of Shi et al. (2020) in their evolutionary multi-task ensemble learning model, who pointed out that efficiently selecting and combining base learners in complex feature spaces remains challenging. Therefore, further research is needed to improve the base learner selection strategy for obtaining optimal combinations.

The multi-strategy combination method of differential evolution algorithm generally performs well, yet it does not guarantee optimal solutions in every instance. In some cases, a single strategy or random combination strategy may lead to better solutions. This finding aligns with the research of Gao et al. (2021), who found that chaotic local search strategies in differential evolution algorithms sometimes outperform complex global search approaches. In large-scale optimization problems, due to time and computational resource constraints, it is impossible to verify all possible strategy combinations exhaustively. Therefore, further optimization of parameter adjustment methods is needed in future research to improve the algorithm’s adaptability and efficiency across a wider range of problems.

Moreover, regarding model interpretability, this study revealed key features and their mechanisms affecting maize variety identification through the SHAP framework. Kumar and Geetha (2022) demonstrated that the computational complexity of SHAP values increases significantly when handling high-dimensional features. Although this study partially addressed this issue through feature selection methods, computational efficiency remains a challenge when processing larger-scale high-dimensional data. Future research could explore more efficient interpretation methods or develop more effective interpretation mechanisms by incorporating domain knowledge to enhance model interpretability and transparency in high-dimensional data applications. Additionally, the interpretability analysis results could be combined with traditional agronomic trait evaluation methods to provide more comprehensive technical support for maize variety breeding and quality improvement.

## 5 Conclusion

This study proposes a multimodal data fusion technique and integrates it with an interpretable stacking ensemble learning model to achieve efficient detection of maize kernel varieties. The base learner is selected and ensemble using diversity and performance indicators, and the discrimination performance is improved using an improved differential evolution algorithm. Finally, the stacking model is explained using the SHAP method. Experimental analysis of the maize kernel variety dataset shows that:

1. The use of multi-modal data fusion techniques can effectively improve the discriminant performance and prediction accuracy of the model. Morphological data selected 15 features as model inputs through RFE, with an accuracy of 52.9%; hyperspectral data selected 106 features as model inputs through SPA, with an accuracy of 90%; and the accuracy was improved to 94.58% by using a feature-level fusion strategy to fuse morphological data and hyperspectral data.
2. Different combinations of base learners have a significant impact on the discrimination performance of the ensemble model. In the selection process, the diversity, accuracy and number of base learners are fully considered, which enhances the stability of the ensemble model and effectively improves the accuracy of the maize kernel variety discrimination results.
3. The adaptive control mechanism of using dynamically adjusted mutation factors and recombination rates, as well as the combination of multiple mutation strategies, improves the differential evolution algorithm. The accuracy, precision, recall and F1 score of the HDE-Stacking ensemble model reached 97.78%, 97.89%, 97.93% and 97.9% respectively, and its stability and comprehensive performance are significantly better than those of other single identification models.
4. The interpretability of the model decision-making process was achieved through the introduction of the SHAP interpretation framework, which revealed the key features that affect the identification of corn varieties: among the hyperspectral features, the 784 nm, 910 nm, 732 nm, 962 nm and 666 nm bands had a significant positive contribution to the recognition results. These bands are mainly related to the protein, fat and carbohydrate content of corn kernels; among the morphological features, ‘a_mean’ (the mean value of the a channel in the color space) was the most influential feature, which is closely related to the apparent characteristics of the kernels. These findings provide an important scientific basis for further optimizing the corn variety recognition system.

In summary, this study not only proposes a high-performance method for identifying corn grain varieties, but also, more importantly, reveals the internal working mechanism of the model decision-making through the SHAP explanation mechanism, providing an interpretable and reliable new path for quickly and accurately identifying corn grain varieties. This interpretable identification method is of great practical significance for guiding corn breeding and variety identification work.

## Data Availability

The raw data supporting the conclusions of this article will be made available by the authors, without undue reservation.
